# Failure to replicate an association of SNPs in the oxidized LDL receptor gene (*OLR1*) with CAD

**DOI:** 10.1186/1471-2350-9-23

**Published:** 2008-04-02

**Authors:** Joshua W Knowles, Themistocles L Assimes, Eric Boerwinkle, Stephen P Fortmann, Alan Go, Megan L Grove, Mark Hlatky, Carlos Iribarren, Jun Li, Richard Myers, Neil Risch, Stephen Sidney, Audrey Southwick, Kelly A Volcik, Thomas Quertermous

**Affiliations:** 1 Division of Cardiovascular Medicine, Falk Cardiovascular Research Building, Stanford University School of Medicine, Stanford, CA, 94305-5406, USA; 2 Department of Health Research and Policy, Redwood Building, Stanford University School of Medicine, Stanford, CA 94305, USA; 3 Stanford Human Genome Center, Department of Genetics, Stanford University School of Medicine, 975 California Ave, Palo Alto, CA, 94304, USA; 4 Institute for Human Genetics, University of California San Francisco, San Francisco, 94143, USA; 5 Division of Research, Kaiser Permanente of Northern California, Oakland, CA, 94612, USA; 6 Stanford Prevention Research Center, Stanford University School of Medicine, Stanford, CA 94305-5705, USA; 7 Human Genetics Center, University of Texas Houston Health Science Center, 1200 Herman Pressler Dr., Houston, TX, 77030, USA; 8 Departments of Epidemiology, Biostatistics and Medicine, University of California, San Francisco, USA

## Abstract

**Background:**

The lectin-like oxidized LDL receptor LOX-1 (encoded by *OLR1*) is believed to play a key role in atherogenesis and some reports suggest an association of *OLR1 *polymorphisms with myocardial infarction (MI). We tested whether single nucleotide polymorphisms (SNPs) in *OLR1 *are associated with clinically significant CAD in the *A*therosclerotic *D*isease, *VA*scular FuNction, & Geneti *C E*pidemiology (ADVANCE) study.

**Methods:**

ADVANCE is a population-based case-control study of subjects receiving care within Kaiser Permanente of Northern California including a subset of participants of the Coronary Artery Risk Development in Young Adults (CARDIA) study. We first resequenced the promoter, exonic, and splice site regions of *OLR1 *and then genotyped four single nucleotide polymorphisms (SNPs), including a non-synonymous SNP (rs11053646, Lys167Asn) as well as an intronic SNP (rs3736232) previously associated with CAD.

**Results:**

In 1,809 cases with clinical CAD and 1,734 controls, the minor allele of the coding SNP was nominally associated with a lower odds ratio (OR) of CAD across all ethnic groups studied (minimally adjusted OR 0.8, P = 0.007; fully adjusted OR 0.8, P = 0.01). The intronic SNP was nominally associated with an increased risk of CAD (minimally adjusted OR 1.12, p = 0.03; fully adjusted OR 1.13, P = 0.03). However, these associations were not replicated in over 13,200 individuals (including 1,470 cases) in the Atherosclerosis Risk in Communities (ARIC) study.

**Conclusion:**

Our results do not support the presence of an association between selected common SNPs in *OLR1 *and the risk of clinical CAD.

## Background

**Figure 1:**
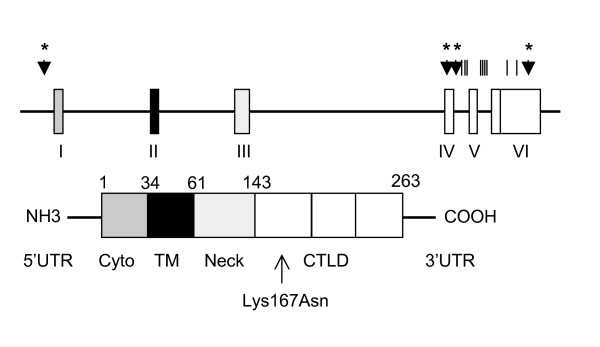
**Structure of LOX-1 gene and protein**. Upper drawing: Rectangles represent exons, with tic marks & arrows designating location of sequenced SNPs and genotyped SNPs in the ADVANCE study respectively. Lower drawing: Schematic of the protein structure of LOX-1. The arrow shows the position of LOX 1.2 SNP, and the resultant amino acid change.

Oxidized LDL (oxLDL) is thought to play a crucial role in the initiation of atherosclerotic lesions. The lectin-like oxidized LDL receptor (LOX-1) is expressed on endothelial cells, macrophages and vascular smooth muscle cells and specifically binds and internalizes oxLDL leading to pleotropic effects on endothelial dysfunction and atherosclerosis. LOX-1 was originally identified by Sawamura et al [1] and was later localized to chromosome 12. The human gene spans six exons and encodes a protein of 273 amino acids with four distinct domains: an N-terminal cytoplasmic domain crucial for cell sorting, a transmembrane domain, a neck domain with homology to the myosin heavy chain, and a C-terminal lectin-like domain (CTLD) which is responsible for binding oxLDL [2-5] (Figure 1). *In vitro*, oxLDL binding to LOX-1 results in increased expression of cellular adhesion molecules, inflammatory mediators and activation of pro-apoptotic pathways [6-10]. *In vivo*, LOX-1 is found at high concentrations in human atherosclerotic lesions and overexpression of LOX-1 in apolipoprotein E -/- mice results in increased cholesterol deposition in coronary arteries [11,12].

Human association studies to date have been conflicting regarding whether polymorphisms in this gene are associated with CAD and/or its complications [13-21]. As part of the overall goals of the *A*therosclerotic *D*isease, *VA*scular FuNction, & Geneti *C E*pidemiology (ADVANCE) study at Stanford University and Kaiser Permanente of Northern California (KPNC), we sought to test whether SNPs in the LOX-1 gene, *ORL1*, alter susceptibility to CAD. We also sought to replicate any putative genotype-phenotype associations in an independent and well characterized cohort as replication is now considered the gold standard method to validate such associations [16,22-27]. We selected the National, Heart, Lung, and Blood Institute's Atherosclerosis Risk in Communities (ARIC) study to serve as our replication cohort because the primary outcome of interest in that study of incident coronary heart disease (CHD) was the same as the ADVANCE study and was diligently adjudicated.

## Methods

### Study Sample ADVANCE

The ADVANCE study included adults (age ≥18 years) receiving medical care within KPNC. Detailed description of the eligibility criteria and the source population for all cohorts, have been published previously [28-30]. The design of ADVANCE allowed for several primary case control comparisons (see study website for details[31]). Between October 28, 2001 and December 31, 2003, we recruited a total of 3179 case and control subjects. Cases consisted subjects presenting with clinically significant CAD (myocardial infarction or angina with angiogram showing at least one > 50% stenosis) at a young age (< 45 years for males, < 55 years for females) or subjects presenting with incident stable angina or incident acute myocardial infarction (AMI) at an older age. Controls consisted of young subjects with no history of CAD including a subset of 479 subjects from the Coronary Artery Risk Development in Young Adults (CARDIA) Study [32], or subjects aged 60 to 72 with no history of CAD, cerebrovascular accident (CVA), or peripheral arterial disease (PAD). During recruitment, some race/ethnic and gender strata were oversampled to maximize the probability that case and control groups were balanced in these respects. In this study, we focused on a combined analysis comparing subjects with symptomatic early onset CAD ("young cases") with young subjects without CAD ("young controls") and older subjects presenting with stable angina or AMI ("older cases") with older subjects with no history of CAD, CVA, or PAD ("older controls") (Table 1).

**Table 1:** Non-genetic characteristics of the ADVANCE study according to case/control status (symptomatic CAD) stratified by two primary predefined comparisons

	**Young Cases **(n = 472)	**Young Controls **(n = 742)	**P**	**Older Cases **(n = 1337)	**Older Controls **(n = 992)	**P**
	
	**Mean(SD)**	**Mean(SD)**	**T test**	**Mean(SD)**	**Mean(SD)**	**T test**
	
Age(years) *	45.3(6.5)	44.3(5.5)	0.005	62(8.4)	65.8(2.9)	<0.001
	
	**Median (Range)**	**Median (Range)**	**Wilcoxon**	**Median (Range)**	**Median (Range)**	**Wilcoxon**
	
**Body Mass Index**	31.1 (17.3–61.2)	26.8 (15.8–66.2)	<0.001	28.4 (16.9–66.1)	27.5 (17.3–52.9)	<0.001
**C-Reactive Protein**	2.3 (0.1–76.4)	1.2 (0.1–76.8)	<0.001	1.7 (0.1–77.4)	1.6 (0.1–73.9)	0.163
**Months from first ever event to study visit**	21.3 (2.7–222.1)†	--	--	3.5 (1.4–26.4)†	--	--
	
	**Count(%)**	**Count(%)**	**Chi^2^**	**Count(%)**	**Count(%)**	**Chi^2^**
	
**Risk Factors (Self Report)**						
**Male**	184(39)	328(44.2)	0.072	975(72.9)	618(62.3)	<0.001
**Current/Former smoker**	276(58.5)	266(35.8)	<0.001	842(63)	569(57.4)	0.006
**Hypertension**	154(32.6)	143(19.3)	<0.001	641(47.9)	407(41)	<0.001
**Diabetes Mellitus**	101(21.4)	51(6.9)	<0.001	274(20.5)	147(14.8)	<0.001
**High Cholesterol**	129(27.3)	148(19.9)	0.003	604(45.2)	356(35.9)	<0.001
**Ancestry**			<0.001			<0.001
**White/European**	254(53.8)	369(49.7)		947(70.8)	677(68.2)	
**Black/African American**	46(9.7)	254(34.2)		50(3.7)	79(8)	
**Hispanic**	25(5.3)	22(3)		83(6.2)	60(6)	
**East Asian**	44(9.3)	35(4.7)		73(5.5)	68(6.9)	
**Admixed Hispanic**	32(6.8)	23(3.1)		52(3.9)	31(3.1)	
**Admixed Non-Hispanics**	71(15)	39(5.3)		132(9.9)	77(7.8)	

Age was defined at first ever event for cases, study visit date for controls. The age cutoff for a "young case" was not the same for males (<45 years) and females (<55 years). †P < 0.001 for Wilcoxon test.

### Clinical Measurements

Through a phone interview, an extensive self administered questionnaire, and the use of the KPNC electronic databases, we documented the presence or absence and age of onset of clinically significant CAD, CVA, and PAD, as well as all traditional risk factors for atherosclerosis. Subjects also provided information on race/ethnicity and were classified into one of nine race/ethnic groups: white/Europeans, black/African Americans, Hispanics, South Asians, East Asians, Pacific Islanders, Native Americans, admixed Hispanics, and admixed non-Hispanics. At the clinic visit, we measured the height and weight of all participants and collected whole blood for DNA extraction and quantification of various serum markers. For this study, traditional risk factors (smoking, hypertension, high cholesterol, and diabetes) were defined based on self report and were considered to be present only if subjects reported an age of onset of a risk factor that was younger than the age of onset of clinically significant CAD.

### Sequencing & Genotyping

Using an automated fluorescent labeling system [33], we resequenced the promoter region, the exons including the 5' and 3' untranslated regions (UTRs), and the intron-exon boundaries of *OLR1 *in 24 ethnically diverse males with a history of CAD (SNP discovery set). A subset of all SNPs identified by sequencing was then genotyped in all participants of the ADVANCE study using the TaqMan^® ^assay. In addition, a random sample of approximately 15% of all genotyped SNPs was genotyped in duplicate. The discrepancy rate among all SNPs that were either sequenced and genotyped or genotyped in duplicate was 0.002%. We were unable to make a genotype call in only 0.35% of the samples. For LOX1.2 and LOX1.3 the reference strand used for the determination of the minor allele was chosen to be consistent with dbSNP convention. Information on all SNPs from the SNP discovery effort has been submitted to the national dbSNP database.

The subset of sequenced SNPs for genotyping in all study participants using the TaqMan^® ^assay [33] was selected on the basis of the minor allele frequency observed in the SNP discovery set, the predicted functional effect, and the degree of linkage disequilibrium between SNPs. In general, we prioritized for genotyping SNPs with a higher MAF (to maximize statistical power), SNPs in exonic or promoter regions (to maximize the probability of a functional effect). When possible, we genotyped SNPs not in strong linkage disequilibrium (LD) with each other to try to identify as many haplotypes as possible across our regions of interest. All resequencing and genotyping was performed by the Stanford Human Genome Center.

### Statistical Analysis

We excluded from further analysis subjects who did not provide blood for DNA extraction (n = 40) and who did not fill out the study questionnaire (n = 9). We also excluded South Asians (n = 55) because of a lack of controls. Lastly, we excluded Pacific Islanders (n = 9) and Native Americans (n = 2) because of small numbers.

For each set of cases and controls, we compared the distributions and frequencies of all non-genetic covariates of interest using standard parametric and non-parametric methods. For each race/ethnic group, we calculated minor allele frequencies of all SNPs genotyped in all participants and tested for Hardy-Weinberg equilibrium (HWE) with the permutation version of the exact test [34]. Using multivariate unconditional logistic regression, we then calculated odds ratios (ORs) for symptomatic CAD associated with the minor allele for each of the three traditional modes of inheritance (recessive, additive, dominant). All ORs were first adjusted for age, gender, self-reported race/ethic group, and source of cases and controls (young set vs old set) and then further adjusted for BMI, smoking status (ever versus never), hypertension, diabetes, and hyperlipidemia. To minimize the probability of confounding due to population substructure in our 'Admixed Hispanic' and 'Admixed Non-Hispanic' race/ethnic groups, we estimated the proportion of white/European, black/African-America, Hispanic and East Asian ancestry at the individual level for all cases and controls in these groups and used these estimates as covariates in our fully adjusted analyses[35]. For each SNP, we also tested for the presence of heterogeneity among the ORs across all race/ethnic groups and both set of cases and controls using appropriate interaction terms in the co-dominant logistic model.

For LOX1.2 and LOX1.3, we calculated the power of our study sample to detect the odds ratios we observed for that SNP. For these calculations, we assumed the polymorphism was causal, a population prevalence of symptomatic CAD of 6.2% [36] and a Type 1 error of 0.05.

We used SAS v 9.1.3 including the SAS genetics module v. 2.2, STRUCTURE[35], and the genetic power calculator [37], to carry out all analyses.

### Replication in the ARIC Study

To avoid reporting spurious findings, it is necessary to replicate putative associations in an independent cohort [22]. Study participants were selected from the ARIC Study, a prospective investigation of atherosclerosis and its clinical sequelae involving 15,792 individuals from four communities aged 45–64 years at recruitment (1987–1989). A detailed description of the ARIC Study design and methods have been published previously [38-43]. Incident coronary heart disease (CHD) cases were defined as definite or probable MI, a silent MI between examinations by ECG, a definite CAD death or coronary revascularization. Genotyping the LOX1.2 SNP (Lys167Asn) was performed using the TaqMan Assay (Applied Biosystems, Foster City, CA) (primers available on request). The LOX1.3 SNP was assayed using a pre-made, validated assay from ABI (assay ID C-3130875-).

Follow-up was available until December 31, 2002. Time-to-event was analyzed using Cox proportional hazards modeling. We adopted the methods of Hsieh and Lavori [44] to calculate post-hoc the minimal detectable hazard ratio based on the standard deviation of genotypes where homozygote major equals 0, heterozygote equals 1 and homozygote minor equals 2.

We used STATA, and PASS [45] to carry out all analyses in the ARIC study.

### Consent and Institutional Review Board Approval

The ADVANCE study was approved by the Institutional Review Boards (IRBs) at Stanford, the Kaiser Foundation Research Institute and the Palo Alto Veterans Administration Hospital. The ARIC study was approved by all participating institutional IRBs. All subjects in both the ADVANCE and ARIC studies gave written informed consent.

## Results

### Non-genetic covariates of interest in ADVANCE

The study population consisted of 3,543 subjects (1,809 cases and 1,734 controls). The prevalence of most traditional risk factors of atherosclerosis was significantly greater in cases than in controls (Table 1). The lower prevalence of males in the younger case/control set and the younger age of cases in the older case/control set were the result of stratified sampling. Stratified sampling and/or preferential participation also led to differences in the prevalence of certain race/ethnic groups.

### LOX1 SNP discovery

We identified 16 SNPs by sequencing the promoter region, the exons and flanking intronic regions of *OLR1 *in the 24 subjects of different ethnic groups from our SNP discovery set (Figure 1). In the promoter region, our SNP discovery primer set started approximately 620 bp 5' from the transcription start site. Three of these SNPs had minor allele frequencies (MAFs) of 2% and were not analyzed further. The remaining SNPs had MAFs of 20% or greater over all race/ethnic groups, and of these SNPs LOX 1.1 (rs2742112), LOX 1.2 (rs11053646), LOX 1.3 (rs3736232) and LOX 1.16 (rs1050286) were selected for genotyping in all participants in the ADVANCE study. Minor allele frequencies for these SNPs are shown in Table 2.

**Table 2:** SNPs in the LOX-1 gene that were genotyped in ADVANCE subjects

**SNP Name**	**dbSNP**	**Type**	**Change**	**Minor/Major Allele**	**Minor Allele Freq (%)**			
					
					**White/European**	**Black/African American**	**East Asian**	**Hispanic**
LOX1.1	rs2742112	5'	391 5' transc start	A/G	31	46	57	39
**LOX1.2**	**rs11053646**	**exon**	**mRNA 563, Lys-Asn**	**C/G**	**9**	**20**	**16**	**6**
LOX1.3	rs3736232	intron	26 3' exon 4	C/G	49	20	23	49
LOX1.16	rs1050286	exon	3' UTR	G/A	49	23	23	50

Bold indicates non-synonymous amino acid change. For association analysis, the minor allele is defined as the least prevalent allele across both sets of controls in all race/ethnic groups combined.

### Association analysis in ADVANCE

We found no evidence of heterogeneity of the ORs across race/ethnic groups and the two sets of cases and controls ("younger" versus "older") for the additive logistic model (details not shown). Therefore, in Tables 3 and 4, we only present the OR for each inheritance model across both sets of cases and controls only stratified by race/ethnic group and combined across race/ethnic groups.

**Table 3:** Genotype counts and ORs for LOX 1.2 in the ADVANCE study combining both sets of cases and controls

				**Recessive**		**Additive**		**Dominant**	
	**Genotype**	**Cases N (%)**	**Controls N (%))**	**minimal model**	**fully adjusted model**	**minimal model**	**fully adjusted model**	**minimal model**	**fully adjusted model**
**LOX1.2**				**OR** **CI**	**OR** **CI**	**OR** **CI**	**OR** **CI**	**OR** **CI**	**OR** **CI**
White	GG	1034 (86)	842 (80)						
	GC	154 (13)	194 (19)	1.44 0.6–3.6	1.59, 0.6–4.0	0.75** 0.6–0.9	0.76* 0.6–0.95	0.69** 0.6–0.9	0.7** 0.6–0.9
	CC	12 (1)	9 (1)						
Black/AA	GG	65 (68)	201 (61)						
	GC	30 (31)	118 (36)	0.25 0.01–1.4	0.23 0.01–1.3	0.8 0.5–1.3	0.77 0.47–1.2	0.85 0.5–1.4	0.82 0.5–1.4
	CC	1 (1)	12 (3)						
Hispanic	GG	96 (89)	73 (89)						
	GC	12 (11)	9 (11)	1 NA	1 NA	1.05 0.4–2.9	1.18 0.4–3.4	1.05 0.4–2.9	1.18 0.4–3.4
	CC	0 (0)	0 (0)						
E. Asian	GG	90 (77)	66 (64)						
	GC	23 (20)	33 (32)	1.02 0.2–4.9	1.94 0.2–4.8	0.61 0.4–1.0	0.56* 0.3–0.98	0.49* 0.3–0.9	0.45* 0.2–0.97
	CC	4 (3)	4 (4)						
Mix. Hisp.	GG	72 (86)	48 (89)						
	GC	11 (13)	6 (11)	3E+09 NA	8E+08 NA	1.66 0.6–5.5	1.51 0.5–5.3	1.64 0.5–5.6	1.51 0.46–5.3
	CC	1 (1)	0 (0)						
Mix. other	GG	150 (74)	92 (79)						
	GC	53 (26)	21 (18)	0* NA	0* NA	1.32 0.8–2.3	1.28 0.73–2.3	1.6 0.9–3.0	1.56 0.8–3.0
	CC	0 (0)	3 (3)						
All	GG	1507 (83)	1322 (76)						
	GC	283 (16)	381 (22)	0.8 0.41–1.5	0.8 0.41–1.5	0.8** 0.7–0.9	0.8* 0.7–0.95	0.77** 0.6–0.93	0.78** 0.7–0.94
	CC	18 (1)	28 (2)						

OR = Odds ratio, ref = reference group, NA = unable to compute, All P values < 0.05 in bold, * P < 0.05, ** P < 0.01.

^† ^combined analyses adjusted for race/ethnic group and case/control set

Minimal model adjusted for age, sex. Fully adjusted analyses adjusted for age, sex, BMI, smoking status, hypertension, diabetes, high cholesterol, "admixed" strata further adjusted for proportion of white, black, Hispanic, E. Asian ancestry derived from STRUCTURE analyses. Non-stratified analyses further adjusted by race/ethnic group and cases/control status.

**Table 4:** Genotype counts and ORs for LOX 1.3 in the ADVANCE study combining both sets of cases and controls

				**Recessive**		**Additive**		**Dominant**	
	**Genotype**	**Cases N (%)**	**Controls N (%))**	**minimal model**	**fully adjusted model**	**minimal model**	**fully adjusted model**	**minimal model**	**fully adjusted model**
**LOX1.3**				**OR** **CI**	**OR** **CI**	**OR** **CI**	**OR** **CI**	**OR** **CI**	**OR** **CI**
White	GG	290 (24)	269 (26)						
	GC	603 (51)	560 (54)	1.29* 1.1–1.6	1.3* 1.1–1.6	1.13 1.0–1.3	1.13 1.0–1.3	1.08 0.88–1.3	1.08 0.88–1.3
	CC	301 (25)	216 (20)						
Black/AA	GG	55 (58)	223 (67)						
	GC	30 (31)	90 (27)	2.31 0.92–5.6	2.5 0.95–6.3	1.47 0.99–2.2	1.49 0.99–2.2	1.49 0.89–2.5	1.49 0.88–2.5
	CC	10 (11)	18 (6)						
Hispanic	GG	43 (30)	20 (24)						
	GC	45 (45)	41 (50)	1.02 0.5–2.1	0.76 0.34–1.7	0.92 0.6–1.4	0.85 0.53–1.4	0.78 0.38–1.6	0.85 0.39–1.8
	CC	19 (25)	21 (26)						
E. Asian	GG	65 (56)	64 (62)						
	GC	44 (38)	35 (34)	2.26 0.6–10	2.56 0.64–12	1.26 0.78–2.0	1.25 0.76–2.1	1.2 0.67–2.2	1.16 0.04–2.1
	CC	7 (6)	4 (4)						
Mix. Hisp.	GG	21 (25)	11 (21)						
	GC	39 (46)	31 (58)	1.39 0.6–3.7	1.45, 0.59–3.7	1.02, 0.59–1.7	1.04 0.6–1.8	0.73 0.29–1.8	0.76 0.3–1.9
	CC	24 (29)	11 (21)						
Mix. other	GG	87 (43)	49 (44)						
	GC	79 (39)	48 (43)	1.01 0.5–2.1	0.98 0.47–2.1	0.42 0.64–1.4	0.93 0.63–1.4	0.85 0.49–1.5	0.86 0.5–1.5
	CC	35 (18)	14 (13)						
All	GG	570 (32)	636 (37)						
	GC	847 (47)	805 (47)	1.3* 1.1–1.6	1.32** 1.1–1.6	1.12* 1.0–1.2	1.13* 1.0–1.25	1.06 0.9–1.25	1.07 0.9–1.25
	CC	380 (21)	284 (16)						

OR = Odds ratio, ref = reference group, NA = unable to compute, All P values < 0.05 in bold, * P < 0.05, ** P < 0.01

^† ^combined analyses adjusted for race/ethnic group and case/control set

Minimal model adjusted for age, sex. Fully adjusted analyses adjusted for age, sex, BMI, smoking status, hypertension, diabetes, high cholesterol, "admixed" strata further adjusted for proportion of white, black, Hispanic, E. Asian ancestry derived from STRUCTURE analyses. Non-stratified analyses further adjusted by race/ethnic group and cases/control status

The LOX 1.1 SNP in the promoter region of the gene was not associated with clinical CAD. However, the "C" allele of the LOX 1.2 SNP (G501C leading to Lys167Asn) was associated with a decreased risk of CAD (MAF of 8.8% in 1808 cases and 11.9% in 1731 controls, fully adjusted OR 0.8, P = 0.01). This association was also significant in a dominant model but not in a recessive model (Table 3). There was no major difference in the OR between the younger set and older set of cases and controls (additive model fully adjusted ORs 0.74, CI 0.55–1.01, p = 0.06 and 0.82, CI 0.67–1.02, P = 0.07 for younger and older case/control set respectively). The "C" allele of the LOX 1.3 SNP was weakly associated with an increased risk of CAD in the combined analysis (MAF of 44.7% in cases and 39.8% in controls, fully adjusted model OR 1.13, P = 0.03) (Table 4). An association with CAD was also present for the recessive model of this SNP (fully adjusted OR 1.32, P = 0.004) but not for a dominant model. There was no major difference in the OR between the younger set and older set of cases and controls (additive model fully adjusted ORs 1.08, CI 0.88–1.32, p = 0.46 and 1.15, CI 1.01–1.32, P = 0.03 for younger and older case/control set respectively). As LOX 1.3 and LOX 1.16 SNPs were found to be in near perfect linkage disequilibrium (details not shown), the results of the association analyses for the LOX 1.16 were essentially identical to LOX1.3 (details not shown).

We performed additional analyses using only the subset of young and old cases presenting with AMI [see Additional file 1]. The results for both SNPs (LOX1.2 and LOX1.3) are not substantially different in any of the three models (recessive, additive or dominant either minimally or fully adjusted) whether cases include all subjects with clinically significant CAD versus cases presenting with MI. While the point estimates for the odds ratios are quite similar there is a loss of significance in the MI comparison presumably because of a decrease in power caused by excluding subjects with incident angina. For instance, LOX 1.2 in our overall analysis has a fully adjusted, additive model OR 0.8 (0.68–0.95, P < 0.05) for the "clinically significant CAD vs control" as compared to an OR 0.84 (0.69–1.02, P = 0.07) for the "MI vs control" comparison. Similar results were found for LOX 1.3 (OR 1.13, 1.0–1.25, P < 0.05 vs 1.08, 0.96–1.22, P = 0.19).

For the least common LOX-1 SNP across all race/ethnic groups combined (LOX1.2), the minimal detectable ORs was 1.2 for an additive model assuming that the minor allele increases risk and 0.71 assuming the minor allele decreases risk.

### Replication in ARIC

Non-genetic characteristics of the ARIC sample are summarized in Table 5. Genotype counts in the ARIC cohort for LOX 1.2 and LOX 1.3 are summarized in Table 6. LOX1.2 was genotyped in a total of 9,858 white and 3,367 African American individuals and the LOX 1.3 SNP was genotyped in 9,844 white and 3,359 African American individuals. After stratification by race, all race/ethnic specific genotype counts were in HWE with the exception of LOX 1.3 which showed some evidence of deviation from HWE in African American subjects (P < 0.01). The MAFs for these SNPs were very similar to that seen in the ADVANCE cohort.

**Table 5:** Characteristics of Incident Coronary Heart Disease cases and non-cases in the Atherosclerosis Risk in Communities cohort, stratitifed by race

	Whites			African Americans		
	Cases	Non-Cases		Cases	Non-Cases	
	(n = 1,168)	(n = 8,802)		(n = 319)	(n = 3,119)	
	
	Mean (SEM)	Mean (SEM)	P**	Mean (SEM)	Mean (SEM)	P**
	
Age (years)^†^	55.7 (0.2)	53.9 (0.06)	<0.0001	55 (0.3)	53.1 (0.1)	<0.0001
Age at event (years)	63.9 (0.2)	--		63.0 (0.4)	--	
Body Mass Index (kg/m2)^†^	27.8 (0.1)	26.8 (0.05)	<0.0001	30 (0.3)	29.5 (0.1)	0.1
HDL Cholesterol (mg/dL)^†^	42.8 (0.4)	52.2 (0.2)	<0.0001	48.6 (0.8)	56.0 (0.3)	<0.0001
Total Cholesterol (mmol/L)^†^	5.82 (0.03)	5.5 (0.01)	<0.0001	5.84 (0.07)	5.51 (0.02)	<0.0001
	
	%	%	P**	%	%	P**
	
Males	69.4	42.2	<0.001	49.5	35.9	<0.001
Diabetes^†^	20.2	6.6	<0.001	34.2	16.4	<0.001
Hypertension^†^	38.6	23.7	<0.001	72.7	52.1	<0.001
Smoking^†^	31.4	23.6	<0.001	42	27.9	<0.001

SEM = Standard error of the mean

*P-value comparing means or proportions between cases and non-cases

^†^At the baseline visit

**Table 6:** Allele frequencies and Hazard Rate Ratios for incident CAD for minor alleles of LOX 1.2, LOX 1.3 in the Atherosclerosis Risk In Communities study

				**Recessive**		**Additive**		**Dominant**	
	**geno**	**Cases**	**Non-cases**	**model 1**	**model 2**	**model 1**	**model 2**	**model 1**	**model 2**
		**counts (%)**	**counts (%)**	**HR** **CI**	**HR** **CI**	**HR** **CI**	**HR** **CI**	**HR** **CI**	**HR** **CI**
**LOX 1.2**									
**Whites**	GG	938 (81)	7075 (81)						
	CG	204 (18)	1549 (18)	1.22 0.7–2.1	1.23 0.7–2.1	1.01 0.88–1.2	0.99 0.86–1.1	1 0.86–1.2	0.97 0.84–1.1
	CC	14 (1)	78 (1)						
	Total	1156	8702						
**AA**	GG	185 (59)	1841 (60)						
	CG	118 (38)	1056 (35)	0.72 0.4–1.3	0.79 0.4–1.4	1 0.83–1.2	0.98 0.8–1.2	1.05 0.84–1.3	1.01 0.8–1.3
	CC	11 (3)	156 (5)						
	Total	314	3053						
**All**	GG	1123 (76)	8916 (76)						
	CG	322 (22)	2065 (18)	0.95 0.64–1.4	1.0 0.7–1.5	1.01 0.9–1.1	0.99 0.88–1.1	1.01 0.9–1.1	0.99 0.88–1.1
	CC	25 (2)	234 (2)						
	Total	1470	11755						
**LOX1.3**									
**Whites**	GG	342 (30)	2393 (27)						
	GC	560 (48)	4329 (50)	0.93 0.81–1.1	0.93 0.81–1.1	0.93 0.86–1.0	0.94 0.87–1.0	0.9, 0.8–1.0	0.91 0.8–1.0
	CC	250 (22)	1970 (23)						
	Total	1152	8692						
**AA**	GG	236 (74)	2099 (69)						
	GC	72 (23)	818 (27)	0.79 0.4–1.5	0.93 0.5–1.8	0.81 0.65–1.0	0.85 0.68–1.1	0.78 0.6–1.0	0.81 0.63–1.0
	CC	10 (3)	124 (4)						
	Total	318	3041						
**All**	GG	578 (39)	4492 (38)						
	GC	632 (43)	5147 (44)	0.92 0.8–1.1	0.93 0.81–1.1	0.92* 0.85–0.99	0.92* 0.86–1.0	0.87* 0.78–0.97	0.89* 0.79–0.99
	CC	260 (18)	2094 (18)						
	Total	1470	11733						

Model 1: adjusted for age and gender (and race in non-stratified analyses).

Model 2: adjusted for age, gender, center, HDL and total cholesterol, BMI, smoking, diabetes and hypertension status (and race in non-stratified analyses). * P < 0.05

Table 6 also summarizes hazard rate ratios (HRR) for symptomatic CHD for the minor allele of each SNP. For LOX1.2, neither the minimally adjusted nor the fully adjusted models demonstrated an association with incident CHD. For LOX1.3, the minor allele showed a nominally significant association with clinical CHD but in the opposite direction than that seen in the ADVANCE study (HRR 0.92, P = 0.04 for the additive model).

We also performed analyses in ARIC in subjects presenting with "incident MI + fatal CHD" as well as "incident non-fatal MI" (see Additional file 1). The results do not differ substantially from those using "incident CHD". For these three comparisons (incident CHD, incident MI + fatal CHD, incident non-fatal MI) the overall HRR, CI and P values in fully adjusted additive model for LOX 1.2 are: 0.99, 0.9–1.1, NS; 0.98, 0.9–1.1, NS; 0.98, 0.8–1.2, NS and for LOX 1.3 are: 0.92, 0.9–1.0, P = 0.04; 0.88, 0.8–1.0, P = 0.01; 0.91, 0.8–1, NS.

The minimal detectable HRR in the ARIC study for LOX1.2 SNP was estimated to be 1.17 for an additive model assuming the minor allele increases risk and 0.85 assuming the minor allele decreases risk.

## Discussion

Oxidized LDL plays a key role in the initiation of atherosclerotic lesions. As an endothelial receptor for oxLDL, LOX-1 has been shown to initiate pro-atherogenic cascades and is an attractive candidate gene for CAD susceptibility [2,4-10,46]. We sought to determine whether SNPs in the LOX-1 gene, *OLR1 *are associated with CAD. In our discovery sample (ADVANCE), we found nominally significant associations of a non-synonymous coding SNP (LOX 1.2) and an intronic SNP (LOX 1.3) with clinical CAD. However, we were unable to replicate these findings in an independent sample (the ARIC study) with adequate power to detect the risk ratio observed in the discovery sample.

Previous human genetic association studies of the LOX1.2 variant (G501C) have yielded conflicting results [13-15,21]. Recently, Morgan et al. were not able to show an association of this SNP with acute coronary syndrome in a study of over 1,400 white subjects [16]. Although the predicted amino acid change (Lys167Asn) occurs in the α1 helix in a region called the "acid-base patch" that might be important for secondary binding of oxLDL [4,5], to our knowledge, there are no reports detailing the effect of the Lys167Asn on oxLDL binding.

Mango et al. studied both the G501C SNP as well as a group of SNPs in introns (rs3736232, rs3736234, rs3736235, rs17174597, rs13306593) and the 3'UTR (rs1050283) of *OLR1 *that were in very strong LD with one another. They found that these non-coding SNPs were highly associated with MI, the OR for MI for carriers of the minor allele at the 3'UTR was 3.7 [14]. This group has recently shown that this group of non-coding SNPs regulates the production of a splice variant of LOX-1 (termed LOXIN), which results in a less robust response to oxLDL and decreased apoptotic signaling. Carriers of the minor alleles of these non-coding SNPs produce less LOXIN, and thus may be at increased risk of CAD [47]. We did genotype one of the SNPs identified by Mango et al. (LOX 1.3, rs3736232) and also found that presence of the minor "C" allele was associated with CAD. However, our efforts to replicate this finding in ARIC were not successful.

In CAD alone, many initially positive reports have not withstood the test of replication in other cohorts The reasons for the frequent failure of candidate gene studies to date to detect robust associations have been widely reviewed [16,48-53].

As in all population genetic studies, the probability of reporting a false negative finding exists. This probability increases significantly in underpowered studies. A key strength of this report is the large number of cases and controls enrolled in the ADVANCE study and the large number of participants in the ARIC study. Thus, based on our power estimates for each study, it is unlikely that we missed a large effect.

Our study has several limitations. First, the ADVANCE study did not enroll cases that either died or were too ill to participate at any time after their incident event and prior to the clinic visit. It is difficult to predict what effect, if any, this selection bias may have had on our observed ORs. Regardless, this selection bias was not present in the ARIC cohort study, which captured incident fatal events. Second, the ARIC study did not have many cases with an age of onset in the same age range as the young cases in the ADVANCE study. Because genotypic effects on CAD risk are generally expected to be greater in subjects with early onset disease [54], a reasonable argument could be made that our inability to replicate associations in the ARIC study was a consequence of a paucity of early onset cases in that cohort. However, the ARIC study still had adequate power to detect the OR noted in the ADVANCE late onset cases and controls for both LOX1.2 and LOX1.3. Furthermore, the nominally significant HRR for LOX1.3 in the opposite direction of the ADVANCE study serves as further compelling evidence that the initial finding was a false positive. Third, because we did not genotype all possible variants or tag SNPs we cannot be certain that other common allelic variants of this gene are not associated with CAD. Based on HapMap data, at least 8 tagged SNPs in white/Europeans and 14 tagSNPs in African Americans (r^2 ^between all SNPs in a bin of ≥ 0.8, minimum minor allele frequency of 1%) would have to be genotyped in this gene to efficiently capture all common haplotypes [55]. However, we did genotype the only validated coding SNP in *OLR1*.

## Conclusion

Large well-powered allelic association studies have great potential for identification of CAD susceptibility genes. The LOX 1.2 and LOX 1.3 SNPs are particularly attractive because the mechanism for risk modification is plausible. We found tentative associations for these SNPs in the ADVACE study. However, all studies like ADVANCE have statistical limitations due to multiple comparisons. To prevent spurious associations, the accepted standard for such analyses is independent replication in another cohort [48-50,56]. We attempted to replicate our findings in the ARIC study, a large community based study of the genetics of atherosclerosis in whites and African Americans. Putative associations must be replicated in multiple large cohorts before they can be assumed to be real. Thus, the overall set of analyses does not support an association between these SNPs and CAD.

## Competing interests

The author(s) declare that they have no competing interests.

## Authors' contributions

JWK and TA were responsible for crucial elements of the study design, data analysis and writing the manuscript. TQ, SPF, MH, AG, CI, RM, NR were responsible for original ADVANCE study including obtaining funding, study design, patient recruitment, genotyping and editing the manuscript. JL and AS were responsible for genotyping efforts and participated in data analysis. SS was the liaison with the CARDIA study and participated in obtaining samples used in the current study. EB, MG and KAV were responsible for the genotyping and data analysis from the ARIC cohort. All authors read and approved the final manuscript.

## Pre-publication history

The pre-publication history for this paper can be accessed here:



## Supplementary Material

Additional file 1 Supplementary tables of LOX1.2 and LOX1.3. These tables detail analysis from both ADVANCE and ARIC specific to myocardial infarction (MI) as an outcome. Click here for file
