# Supplementary material for: Failure to replicate an association of SNPs in the oxidized LDL receptor gene (OLR1) with CAD
Source: BMC Med Genet. 2008 Apr 2;9:23. doi: 10.1186/1471-2350-9-23 (PMC2322963; doi:10.1186/1471-2350-9-23)
Supplement: Additional file 1 — Supplementary tables of LOX1.2 and LOX1.3. These tables detail analysis from both ADVANCE and ARIC specific to myocardial infarction (MI) as an outcome. [file 1471-2350-9-23-S1.doc]

**Failure to replicate an association of SNPs in the oxidized LDL receptor gene (*OLR1*) with CAD**

SUPPLEMENT

Joshua W. Knowles^1*^, Themistocles L. Assimes^1*^, Eric Boerwinkle^7^, Stephen P. Fortmann^6^, Alan Go^5^, Megan L. Grove^7^, Mark Hlatky^1,2^, Carlos Iribarren^5^, Jun Li^3^, Richard Myers^3^, Neil Risch^4,5,8^, Stephen Sidney^5^, Audrey Southwick^3^, Kelly A. Volcik^7^, Thomas Quertermous^1^

^1^ Division of Cardiovascular Medicine, Falk Cardiovascular Research Building, Stanford University School of Medicine, Stanford, CA, 94305-5406, USA

^2^ Department of Health Research and Policy, Redwood Building, Stanford University School of Medicine, Stanford, CA 94305,USA

^3^ Stanford Human Genome Center, Department of Genetics, Stanford University School of Medicine, 975 California Ave, Palo Alto, CA, 94304, USA

^4^ Institute for Human Genetics, University of California San Francisco, San Francisco, 94143, USA

^5^ Division of Research, Kaiser Permanente of Northern California, Oakland, CA, 94612, USA

^6^ Stanford Prevention Research Center, Stanford University School of Medicine, Stanford, CA 94305-5705, USA

^7^ Human Genetics Center, University of Texas Houston Health Science Center, 1200 Herman Pressler Dr., Houston, TX, 77030, USA

^8^ Departments of Epidemiology, Biostatistics and Medicine, University of California, San Francisco, USA

* these authors contributed equally to this work

**Table 1s.** **Genotype counts and ORs for LOX 1.2 in the ADVANCE study combining both sets of cases (MI only) and controls**

|  |  |  |  | **Recessive** | | **Additive** | | **Dominant** | |
| --- | --- | --- | --- | --- | --- | --- | --- | --- | --- |
|  | **Geno-type** | **Cases**  **N (%)** | **Controls**  **N (%))** | **minimal**  **model** | **fully**  **adjusted**  **model** | **minimal**  **model** | **fully**  **adjusted**  **model** | **minimal**  **model** | **fully**  **adjusted**  **model** |
|  |  |  |  |  |  |  |  |  |  |
| **LOX1.2** |  |  |  | **OR**  **CI** | **OR**  **CI** | **OR**  **CI** | **OR**  **CI** | **OR**  **CI** | **OR**  **CI** |
| White | GG | 658 (85) | 842 (80) |  |  |  |  |  |  |
|  | GC | 109 (14) | 194 (19) | 1.54  0.5-4.3 | 1.7  0.6-4.7 | 0.81  0.7-1.0 | 0.84  0.6-1.1 | **0.77**  **0.6-1.0*** | 0.79  0.6-1.0) |
|  | CC | 7 (1) | 9 (1) |  |  |  |  |  |  |
| Black/AA | GG | 50 (70) | 201 (61) |  |  |  |  |  |  |
|  | GC | 20 (28) | 118 (36) | 0.33 0.02-1.9 | 0.34  0.02-2.0 | 0.77  0.5-1.3 | 0.76  0.4-1.3 | 0.80  0.4-1.4 | 0.79  0.4-1.4 |
|  | CC | 1 (1) | 12 (3) |  |  |  |  |  |  |
| Hispanic | GG | 61 (90) | 73 (89) |  |  |  |  |  |  |
|  | GC | 7 (10) | 9 (11) | 1.0  NA | 1.0  NA | 0.85  0.3-2.7 | 0.78  0.2-2.6 | 0.85  0.3-2.7 | 0.78  0.2-2.6 |
|  | CC | 0 (0) | 0 (0) |  |  |  |  |  |  |
| E. Asian | GG | 55 (73) | 66 (64) |  |  |  |  |  |  |
|  | GC | 18 (24) | 33 (32) | 0.78  0.1-5.1 | 0.72  0.1-4.9 | 0.68  0.4-1.3 | 0.63  0.3-1.2 | 0.63  0.3-1.3 | 0.58  0.3-1.2 |
|  | CC | 2 (3) | 4 (4) |  |  |  |  |  |  |
| Mix.Hisp. | GG | 46 (90) | 48 (89) |  |  |  |  |  |  |
|  | GC | 4 (8) | 6 (11) | 4E+09  NA | 8E+09  NA | 0.99  0.3-3.7 | 0.80  0.2-3.5 | 0.90  0.2-3.7 | 0.76  0.2-3.4 |
|  | CC | 1 (2) | 0 (0) |  |  |  |  |  |  |
| Mix.other | GG | 97 (75) | 92 (79) |  |  |  |  |  |  |
|  | GC | 33 (25) | 21 (18) | 0  NA | 0  NA | 1.3  0.7-2.4 | 1.3  0.7-2.5 | 1.58  0.8-3.1 | 1.63  0.8-3.3 |
|  | CC | 0 (0) | 3 (3) |  |  |  |  |  |  |
| All | GG | 967 (83) | 1322 (76) |  |  |  |  |  |  |
|  | GC | 191 (16) | 381 (22) | 0.79  0.4-1.6 | 0.81,  0.4-1.7 | **0.82**  **0.7-1.0*** | 0.84  0.69-1.0 | 0.81  0.66-0.99 | 0.82  0.67-1.0 |
|  | CC | 11 (1) | 28 (2) |  |  |  |  |  |  |

OR = Odds ratio, ref = reference group, NA = unable to compute, All P values < 0.05 in bold, * P < 0.05, ** P < 0.01

^†^ combined analyses adjusted for race/ethnic group and case/control set

Minimal model adjusted for age, sex. Fully adjusted analyses adjusted for age, sex, BMI, smoking status, hypertension, diabetes, high cholesterol, “admixed” strata further adjusted for proportion of white, black, Hispanic, E. Asian ancestry derived from STRUCTURE analyses. Non-stratified analyses further adjusted by race/ethnic group and cases/control status.

**Table 2s.** **Genotype counts and ORs for LOX 1.3 in the ADVANCE study combining both sets of cases (MI only) and controls**

|  |  |  |  | **Recessive** | | **Additive** | | **Dominant** | |
| --- | --- | --- | --- | --- | --- | --- | --- | --- | --- |
|  | **Geno-type** | **Cases**  **N (%)** | **Controls**  **N (%))** | **minimal**  **model** | **fully**  **adjusted**  **model** | **minimal**  **model** | **fully**  **adjusted**  **model** | **minimal**  **model** | **fully**  **adjusted**  **model** |
|  |  |  |  |  |  |  |  |  |  |
| **LOX1.3** |  |  |  | **OR**  **CI** | **OR**  **CI** | **OR**  **CI** | **OR**  **CI** | **OR**  **CI** | **OR**  **CI** |
| White | GG | 197 (26) | 269 (26) |  |  |  |  |  |  |
|  | GC | 386 (50) | 560 (54) | 1.18  0.9-1.5 | 1.18  0.9-1.5 | 1.06  0.9-1.2 | 1.06  0.9-1.2 | 1.01  0.8-1.3 | 1.0, 0.8-1.3 |
|  | CC | 186 (24) | 216 (20) |  |  |  |  |  |  |
| Black/AA | GG | 37 (52) | 223 (67) |  |  |  |  |  |  |
|  | GC | 25 (36) | 90 (27) | 2.23  0.8-5.7 | 2.51  0.9-6.9 | 1.56  1.0-2.4 | 1.59  1.0-2.5 | 1.67  1.0-2.9 | 1.66  0.9-3.0 |
|  | CC | 8 (11) | 18 (6) |  |  |  |  |  |  |
| Hispanic | GG | 19 (29) | 20 (24) |  |  |  |  |  |  |
|  | GC | 30 (46) | 41 (50) | 0.88  0.4-2.0 | 0.67  0.3-1.6 | 0.85  0.5-1.4 | 0.75  0.4-1.3 | 0.72  0.3-1.6 | 0.70  0.3-1.7 |
|  | CC | 16 (25) | 21 (26) |  |  |  |  |  |  |
| E. Asian | GG | 41 (55) | 64 (62) |  |  |  |  |  |  |
|  | GC | 28 (37) | 35 (34) | 3.2  0.75-16 | 3.56  0.8-19 | 1.39  0.8-2.4 | 1.40  0.8-2.5 | 1.28  0.7-2.5 | 1.27  0.7-2.5 |
|  | CC | 6 (8) | 4 (4) |  |  |  |  |  |  |
| Mix.Hisp. | GG | 14 (27) | 11 (21) |  |  |  |  |  |  |
|  | GC | 22 (43) | 31 (58) | 1.50  0.6-4.0 | 1.54  0.6-4.3 | 1.0  0.6-1.8 | 1.02  0.6-1.9 | 0.65  0.2-1.7 | 0.68  0.2-1.9 |
|  | CC | 15 (29) | 11 (21) |  |  |  |  |  |  |
| Mix.other | GG | 58 (45) | 49 (44) |  |  |  |  |  |  |
|  | GC | 49 (38) | 48 (43) | 0.97  0.4-2.2 | 0.92  0.4-2.1 | 0.92  0.6-1.4 | 0.91  0.6-1.4 | 0.86  0.5-1.5 | 0.87  0.5-1.6 |
|  | CC | 21 (16) | 14 (13) |  |  |  |  |  |  |
| All | GG | 366 (32) | 636 (37) |  |  |  |  |  |  |
|  | GC | 540 (47) | 805 (47) | 1.21  1.0-1.5 | 1.22  1.0-1.5 | 1.08  1.0-1.21 | 1.08  1.0-1.2 | 1.03  0.9-1.2 | 1.03  0.9-1.2 |
|  | CC | 252 (22) | 284 (16) |  |  |  |  |  |  |

OR = Odds ratio, ref = reference group, NA = unable to compute, All P values < 0.05 in bold, * P < 0.05, ** P < 0.01

^†^ combined analyses adjusted for race/ethnic group and case/control set

# Minimal model adjusted for age, sex. Fully adjusted analyses adjusted for age, sex, BMI, smoking status, hypertension, diabetes, high cholesterol, “admixed” strata further adjusted for proportion of white, black, Hispanic, E. Asian ancestry derived from STRUCTURE analyses. Non-stratified analyses further adjusted by race/ethnic group and cases/control status

# Table 3s. Allele frequencies and Hazard Rate Ratios for incident MI and fatal CHD for minor alleles of LOX 1.2, LOX 1.3 in the Atherosclerosis Risk In Communities study

|  |  |  |  | **Recessive** | | **Additive** | | **Dominant** | |
| --- | --- | --- | --- | --- | --- | --- | --- | --- | --- |
|  | **geno** | **Cases** | **Non-cases** | **model**  **1** | **model 2** | **model**  **1** | **model 2** | **model**  **1** | **model**  **2** |
|  |  | **counts (%)** | **counts (%)** | **HR**  **CI** | **HR**  **CI** | **HR**  **CI** | **HR**  **CI** | **HR**  **CI** | **HR**  **CI** |
| **LOX 1.2** |  |  |  |  |  |  |  |  |  |
| **Whites** | GG | 551 (83) | 7462 (81) |  |  |  |  |  |  |
|  | CG | 109 (16) | 1644 (18) | 1.37  0.7-2.7 | 1.38  0.7-2.7 | 0.95  0.8-1.1 | 0.93  0.8-1.1 | 0.92  0.8-1.1 | 0.90  0.7-1.1 |
|  | CC | 9 (1) | 83 (1) |  |  |  |  |  |  |
|  | Total | 669 | 9189 |  |  |  |  |  |  |
| **AA** | GG | 145 (57) | 1881 (60) |  |  |  |  |  |  |
|  | CG | 98 (39) | 1076 (35) | 0.82  0.4-1.6 | 0.92  0.5-1.7 | 1.06  0.9-1.3 | 1.05  0.9-1.3 | 1.12  0.9-1.4 | 1.1  0.9-1.3 |
|  | CC | 10 (4) | 157 (5) |  |  |  |  |  |  |
|  | Total | 253 | 3114 |  |  |  |  |  |  |
| **All** | GG | 696 (75) | 9343 (76) |  |  |  |  |  |  |
|  | CG | 207 (22) | 2720 (22) | 1.03  0.7-1.6 | 1.1  0.7-1.7 | 1.0  0.9-1.1 | 0.98  0.9-1.1 | 0.99  0.9-1.2 | 0.96  0.8-1.1 |
|  | CC | 19 (2) | 240 (2) |  |  |  |  |  |  |
|  | Total | 922 | 12303 |  |  |  |  |  |  |
| **LOX1.3** |  |  |  |  |  |  |  |  |  |
| **Whites** | GG | 212 (31) | 2523 (27) |  |  |  |  |  |  |
|  | GC | 314 (47) | 4575 (50) | 0.93  0.8-1.1 | 0.91  0.8-1.1 | 0.90  0.8-1.0 | 0.90  0.8-1.0 | **0.82***  0.7-1.0 | **0.83***  0.7-1.0 |
|  | CC | 145 (22) | 2075 (23) |  |  |  |  |  |  |
|  | Total | 671 | 9173 |  |  |  |  |  |  |
| **AA** | GG | 191 (75) | 2144 (69) |  |  |  |  |  |  |
|  | GC | 59 (23) | 831(27) | 0.58  0.3-1.3 | 0.70  0.3-1.6 | **0.77***  0.6-1.0 | 0.81  0.6-1.0 | 0.76  0.6-1.0 | 0.79  0.6-1.1 |
|  | CC | 6 (2) | 128 (4) |  |  |  |  |  |  |
|  | Total | 256 | 3103 |  |  |  |  |  |  |
| **All** | GG | 403 (43) | 4667 (38) |  |  |  |  |  |  |
|  | GC | 373 (40) | 5406 (44) | 0.9  0.8-1.1 | 0.90  0.8-1.1 | **0.88***  0.8-1.0 | **0.88***  0.8-1.0 | **0.81***  0.7-0.9 | **0.82***  0.7-0.9 |
|  | CC | 151 (16) | 2203 (18) |  |  |  |  |  |  |
|  | Total | 927 | 12276 |  |  |  |  |  |  |

Model 1: adjusted for age and gender (and race in non-stratified analyses).

Model 2: adjusted for age, gender, center, HDL and total cholesterol, BMI, smoking, diabetes and hypertension status (and race in non-stratified analyses). * P < 0.05

# Table 4s. Allele frequencies and Hazard Rate Ratios for incident non-fatal MI for minor alleles of LOX 1.2, LOX 1.3 in the Atherosclerosis Risk In Communities study

|  |  |  |  | **Recessive** | | **Additive** | | **Dominant** | |
| --- | --- | --- | --- | --- | --- | --- | --- | --- | --- |
|  | **geno** | **Cases** | **Non-cases** | **model**  **1** | **model 2** | **model**  **1** | **model 2** | **model**  **1** | **model**  **2** |
|  |  | **counts (%)** | **counts (%)** | **HR**  **CI** | **HR**  **CI** | **HR**  **CI** | **HR**  **CI** | **HR**  **CI** | **HR**  **CI** |
| **LOX 1.2** |  |  |  |  |  |  |  |  |  |
| **Whites** | GG | 426 (82) | 7538 (81) |  |  |  |  |  |  |
|  | CG | 86 (17) | 1656 (18) | 0.99  0.4-2.4 | 1.0  0.4-2.4 | 0.93  0.8-1.2 | 0.91  0.7-1.1 | 0.92  0.7-1.2 | 0.90  0.7-1.1 |
|  | CC | 5 (1) | 86 (1) |  |  |  |  |  |  |
|  | Total | 517 | 9280 |  |  |  |  |  |  |
| **AA** | GG | 91 (57) | 1917 (60) |  |  |  |  |  |  |
|  | CG | 63 (39) | 1102 (35) | 0.90  0.4-1.9 | 1.02  0.5-2.2 | 1.09  0.8-1.4 | 1.09  0.8-1.4 | 1.16  0.9-1.6 | 1.12  0.8-1.5 |
|  | CC | 7 (4) | 158 (5) |  |  |  |  |  |  |
|  | Total | 161 | 3177 |  |  |  |  |  |  |
| **All** | GG | 517 (76) | 9455 (76) |  |  |  |  |  |  |
|  | CG | 149 (22) | 2758 (22) | 0.95  0.5-1.7 | 1.01  0.6-1.8 | 0.99  0.8-1.2 | 0.98,  0.8-1.2 | 0.99  0.8-1.2 | 0.97  0.8-1.2 |
|  | CC | 12 (2) | 244 (2) |  |  |  |  |  |  |
|  | Total | 678 | 12457 |  |  |  |  |  |  |
| **LOX1.3** |  |  |  |  |  |  |  |  |  |
| **Whites** | GG | 157 (30) | 2556 (27) |  |  |  |  |  |  |
|  | GC | 250 (48) | 4613 (50) | 0.93  0.8-1.2 | 0.93  0.8-1.2 | 0.93  0.8-1.1 | 0.93  0.8-1.1 | 0.88  0.7-1.1 | 0.89  0.7-1.1 |
|  | CC | 113 (22) | 2094 (23) |  |  |  |  |  |  |
|  | Total | 520 | 9263 |  |  |  |  |  |  |
| **AA** | GG | 122 975) | 2193 (69) |  |  |  |  |  |  |
|  | GC | 36 (22) | 845 (27) | 0.77  0.3-1.9 | 0.90  0.4-2.2 | 0.79,  0.6-1.1 | 0.83  0.6-1.2 | 0.88  0.7-1.1 | 0.79  0.6-1.1 |
|  | CC | 5 (3) | 128 (4) |  |  |  |  |  |  |
|  | Total | 163 | 3166 |  |  |  |  |  |  |
| **All** | GG | 279 (41) | 4749 (38) |  |  |  |  |  |  |
|  | GC | 286 (42) | 5458 (44) | 0.92  0.8-1.1 | 0.93  0.8-1.1 | 0.91  0.8-1.0 | 0.91  0.8-1.0 | 0.85  0.7-1.0 | 0.86  0.7-1.0 |
|  | CC | 118 (17) | 2222 (18) |  |  |  |  |  |  |
|  | Total | 683 | 12429 |  |  |  |  |  |  |

Model 1: adjusted for age and gender (and race in non-stratified analyses).

Model 2: adjusted for age, gender, center, HDL and total cholesterol, BMI, smoking, diabetes and hypertension status (and race in non-stratified analyses). * P< 0.05
